# Intrinsic connectivity within the affective salience network moderates adolescent susceptibility to negative and positive peer norms

**DOI:** 10.1038/s41598-022-17780-1

**Published:** 2022-10-19

**Authors:** Kathy T. Do, Ethan M. McCormick, Mitchell J. Prinstein, Kristen A. Lindquist, Eva H. Telzer

**Affiliations:** 1 Department of Psychology and Neuroscience, University of North Carolina Chapel Hill, 235 E. Cameron Avenue, Chapel Hill, NC 27599-3270 USA; 2 Cognitive Neuroscience Department, Donders Institute for Brain, Cognition and Behavior, Radboud University Medical Center, Nijmegen, The Netherlands

**Keywords:** Cognitive neuroscience, Risk factors

## Abstract

Not all adolescents are equally susceptible to peer influence, and for some, peer influence exerts positive rather than negative effects. Using resting-state functional magnetic resonance imaging, the current study examined how intrinsic functional connectivity networks associated with processing social cognitive and affective stimuli predict adolescents’ (*n* = 87, ages 11–14 years) prosocial tendencies and risky behaviors in the context of positive and negative peer norms. We tested the moderating role of four candidate intrinsic brain networks—associated with mentalizing, cognitive control, motivational relevance, and affective salience—in peer influence susceptibility. Only intrinsic connectivity within the affective salience network significantly moderated the association between peer norms and adolescent behavior above and beyond the other networks. Adolescents with high intrinsic connectivity within the affective salience network reported greater prosocial tendencies in contexts with more positive peer norms but greater risk-taking behavior in contexts with more negative peer norms. In contrast, peer norms were not associated with adolescent behavior for individuals with low affective salience within-network intrinsic connectivity. The mentalizing network, cognitive control network, and motivational relevance network were not associated with individual differences in peer influence susceptibility. This study identifies key neural mechanisms underlying differential susceptibility to positive and negative peer influence in early adolescence, with a particular emphasis on the role of affective salience over traditional mentalizing, regulatory, and motivational processes.

## Introduction

On average, adolescents are more susceptible to peer influence than other age groups—in the presence of risky peer norms, adolescents are more likely to engage in negative behaviors and in the presence of prosocial peer norms, adolescents are more likely to engage in positive behaviors^1,2^. Nonetheless, there is variability in this susceptibility, with some adolescents being highly sensitive to conformity demands whereas others are remarkably resistant^3^. What leads some adolescents to be more susceptible—for better, or worse—and some to be more impassive to the peer context? During adolescence, brain regions involved in processing social cognitive and affective information show significant changes in structure^4^, function^5^, and connectivity^6^ and are robustly recruited during social decision making^7^. At the same time, adolescents start to spend more time with peers, who offer them unique opportunities to learn about social norms^8^ and develop social cognition^9^. These psychosocial processes interact with puberty-induced changes in brain maturation to further orient adolescents towards their peers and increase the importance of social rewards and punishments^10^. These changes make brain networks associated with processing social cognitive and affective stimuli important targets of investigation. Few studies, however, have taken a comprehensive approach to characterize the role of multiple social, cognitive, and affective brain networks in adolescent peer influence. Our first goal was to examine how four intrinsic brain networks important for social, cognitive, and affective processing—referred to here as the mentalizing, cognitive control, motivational relevance, and affective salience networks—might interact with the peer context to predict adolescents’ prosocial tendencies and risk-taking behaviors.

### The role of social cognitive and affective networks in adolescent behavior

First, midline/lateral cortical regions within the “mentalizing” network are associated with perceiving and interpreting the mental states of others (Table 1). Neural responses within the mentalizing network in the presence of peers or in anticipation of peer feedback have been linked to individual differences in rejection sensitivity^11^ and peer conformity to prosocial and risky behaviors^12,13^. Second, the frontoparietal “cognitive control” network is associated with self-regulation and goal-directed behavior (Table 1). Although peer influence has previously been associated with immature cognitive control functioning^14^, emerging research suggests that peers can incentivize a flexible implementation of cognitive control to support goal-directed behaviors^15^. Third, the dopaminergic “motivational relevance” network is associated with gaining rewards^16^ and avoiding punishments^17^ (Table 1). For adolescents but not adults, the presence of peers increases activity within the motivational relevance network during reward processing and risk-taking tasks^1^. Finally, the “affective salience” network is associated with the experience of pleasant or unpleasant feelings^18^, social rejection^19^ and vigilance towards stimuli of socio-emotional significance (Table 1). Compared to older adolescents, younger adolescents show greater activation within midcingulo-insular regions that comprise the affective salience network when exposed to social rejection^20^.

**Table 1:** Proposed intrinsic brain networks relevant for peer influence susceptibility.

A priori network	Regions	Function
Mentalizing	Temporoparietal junction, posterior superior temporal sulcus, posterior cingulate, and temporal poles, dorsal medial prefrontal cortex^21,22^	Perception and interpretation of the mental states of others
Cognitive control	Dorsolateral prefrontal cortex, inferior parietal lobe^23,24^	Goal-driven initiation and inhibition of behavioral responses
Motivational relevance	Ventral striatum, caudate, ventral tegmental area, orbitofrontal cortex^25^	Motivation to gain social reward and avoid social punishment
Affective salience	Amygdala, dorsal anterior cingulate cortex, anterior insula, putamen^26,27^	Vigilance toward stimuli involving social threat or reward

Importantly, these four networks not only show greater functional connectivity during social cognitive and affective tasks^28–30^, but are also identifiable as intrinsic connectivity networks present even when participants are not engaged in an external task and are in a so-called “resting state”^31^. Intrinsic brain networks are thought to reflect relatively stable functional connectivity patterns that may predispose a person to a particular type of cognitive or affective state^29^, making them promising markers of adolescents’ general susceptibility to the peer environment. We hypothesized that greater connectivity within all four intrinsic networks may reflect individuals who have greater social cognitive and affective processing of the broader peer environment. Specifically, highly susceptible adolescents may exhibit greater intrinsic functional connectivity (1) within the mentalizing network associated with heightened consideration of peers’ mental states^13^; (2) within the cognitive control network associated with heightened flexibility in deploying cognitive resources to meet social goals^15^; (3) within the motivational relevance network associated with heightened motivation to seek social rewards and avoid social punishments^32^; and (4) within the affective salience network associated with heightened vigilance to social acceptance and rejection^33^. It is also possible that functional connectivity within one network may predispose adolescents to peer influence more so than others.

### Network connectivity and differential susceptibility to peer contexts

The differential susceptibility theory^34,35^ explains how individual differences in social cognitive and affective sensitivity might moderate the association between peer influence and adolescent behavior. The differential susceptibility theory suggests that an individual’s general susceptibility interacts with the valence of the social context to predict developmental outcomes. We thus hypothesized that intrinsic connectivity within social cognitive and affective networks will serve as a susceptibility marker, increasing adolescents’ sensitivity to their peer environment, and rendering those adolescents more likely to endorse both risky and prosocial behaviors when the peer context promotes those behaviors. Insofar as regions within each candidate network are associated with both healthy and unhealthy developmental outcomes, we predict that in a for-better *and* for-worse manner, youth with high levels of intrinsic connectivity within social cognitive and affective networks will be both more vulnerable to negative peer influence and more sensitive to positive peer influence compared to their less susceptible counterparts. Although we hypothesized that all four social cognitive and affective networks would contribute to individual differences in peer influence susceptibility, our second goal was to identify which networks might be a stronger predictor of heightened susceptibility to the peer context.

Our prior work shows that adolescents’ sensitivity to social rewards and punishments in the ventral striatum serves as a susceptibility factor that interacts with deviant peer norms to predict adolescent risk taking^36^. However, this study could not examine whether highly neurobiologically susceptible youth may also reap beneficial outcomes when they encounter positive peer environments. Furthermore, given several social, cognitive, and affective brain regions collectively support social decision making in adolescence^7^, it is critical to examine whether there is specificity in the type of intrinsic brain networks that moderate peer influence susceptibility, potentially reflecting domain-general mechanisms that predispose some adolescents to be highly sensitive to acute peer influences.

### Current study

Using a cross-sectional sample of early adolescents (*n* = 87, 11–14 years old), the current study examined how intrinsic functional connectivity networks associated with processing social cognitive and affective stimuli predicted adolescents’ endorsement of prosocial and risky behaviors in the context of positive and negative peer norms. To this end, we tested the roles of four candidate intrinsic brain networks in moderating links between peer norms and adolescent behavior: mentalizing, cognitive control, motivational relevance, and affective salience networks. Drawing on the differential susceptibility theory, we hypothesized that adolescents with high intrinsic connectivity within these social cognitive and affective networks would endorse more prosocial behaviors in the context of positive peer norms and more risk-taking behaviors in the context of negative peer norms. In contrast, adolescents with lower intrinsic connectivity within these networks would be less affected by their peer norms.

## Methods

### Participants

The current study is part of a larger longitudinal functional magnetic resonance imaging (fMRI) study. Participants were recruited from a study of 873 6th- and 7th-grade students from a rural community in the Southeastern United States. Exclusion criteria included learning disabilities, braces, head trauma, or other MRI contraindications. Adolescent participants whose primary guardian reported their adolescent was currently using psychoactive medications were instructed to abstain from use for a minimum of 24 h before the visit. A total of 139 participants were enrolled in the longitudinal neuroimaging study. An additional nine participants were recruited but met exclusion criteria at their baseline visit (*n* = 6 could not complete the MRI scan (e.g., claustrophobia, non-compliant), *n* = 1 had metal implant, *n* = 1 was not fluent in English, and *n* = 1 had a brain abnormality). The study protocol was approved by the University of North Carolina at Chapel Hill’s Institutional Review Board. All research was performed in accordance with relevant guidelines and regulations. The primary guardian provided informed consent and adolescent participants provided informed assent prior to the study.

Of the 139 original participants, 110 participants completed an MRI scan at the first two waves (*n* = 13 did not want to participate again (e.g., change of residence, loss of interest), *n* = 8 were difficult to schedule or contact for their follow-up visit, *n* = 8 completed behavioral measures but could not complete an MRI scan at their follow-up visit due to braces or other metal implants). The current study reports data from only the first wave of data, as the longitudinal resting-state MRI (rsfMRI) effects are the focus of a separate manuscript. Time constraints at the scan visit sometimes precluded collection of rsfMRI data in the full sample. 19 participants were missing rsfMRI, 1 participant was missing questionnaire data, and 3 participants had poor quality rsfMRI data (e.g., framewise displacement (FD) > 0.30 mm on > 10% volumes before scrubbing, < 4 min of data acquisition), resulting in a total sample of 87 participants (*M*_*age*_ = 12.84 years, *SD*_*age*_ = 0.53 years, *range* = 11.94–14.49 years, 49 female). The race/ethnicity of the final sample included: White (*n* = 26, 29.9%), Black (*n* = 21, 24.1%), Latinx (*n* = 30, 34.5%), Multi-racial (*n* = 8, 9.2%), and Other (*n* = 2, 2.3%). The primary guardian reported their highest level of education as: less than high school (*n* = 19, 21.8%), high school (*n* = 14, 16.1%), some college (*n* = 27, 31%), associate’s degree (*n* = 15, 17.2%), bachelor’s degree (*n* = 5, 5.7%), some graduate school (*n* = 3, 3.4%), master’s degree (*n* = 2, 2.3%), and professional degree (*n* = 2, 2.3%). Results using task-based fMRI measures of peer influence susceptibility from the first wave have previously been published^36^.

### Procedure

Participants completed a 1.5 h fMRI scan, which included structural sequences, five task-based sequences that are not the focus of the current manuscript, and the resting-state sequence (described below). To minimize head motion and increase awareness of small movements during the scan, foam padding was placed around adolescents’ heads and masking tape was placed across adolescents’ foreheads. After the scan, adolescents reported on their peer group norms and endorsement of prosocial and risk-taking behaviors using Qualtrics online survey software, as well as completed other self-report measures and behavioral tasks which are not the focus of this manuscript. The primary guardian completed a series of self-report measures that are not the focus of the current manuscript. For their participation, adolescents were compensated $90 cash, a meal, and small non-monetary prizes for completing the full fMRI scan and staying still (e.g., headphones, candy; $20 value). Primary guardians were compensated $50 cash, a meal, $20 gas, and parking.

### Questionnaire measures

#### Perceived peer group norms

To test the effect of the valence of the peer context, we measured negative and positive peer influence. Perceived peer group norms were assessed using a revised version of the Perception of Peer Group Norms Questionnaire^37^, in which adolescents responded to 16 questions indicating how many of their close friends engage in negative behaviors and positive behaviors in general (1 = *none* to 6 = *almost all*). Scores were computed as the mean rating of the negative and (reverse coded) positive items and standardized. Lower scores indicate relatively more positive than negative peer group norms, higher scores indicate relatively more negative than positive peer group norms, and scores of 0 indicate relatively equal levels of positive and negative peer group norms (Cronbach alpha = 0.83).

#### Adolescent behavior

To test whether highly susceptible youth can be simultaneously susceptible to peer contexts in a for-better and for-worse manner, we measured adolescents’ endorsement of prosocial and risk-taking behaviors.

##### Prosocial tendencies

Prosocial tendencies were assessed using the Prosocial Tendencies Measure^38^, in which adolescents indicated their endorsement of various prosocial behaviors (e.g., “I tend to help others in need when they do not know who helped them”) (1 = *does not describe me at all* to 5 = *describes me greatly*). Scores were computed as the mean rating of the 21 items, with higher scores reflecting higher prosocial tendencies (Cronbach alpha = 0.75).

##### Risk-taking behavior

The frequency of risk-taking behavior was assessed using a revised Adolescent Risk Taking Scale^39^, in which adolescents reported the number of times they engaged in health risk or deviant behaviors (e.g., “I have willingly ridden in a car with someone I knew was a dangerous driver”) (0 = *never* to 3 = *many times*). Scores were computed as the mean rating of the 14 items, with higher scores reflecting higher frequency of risk taking (Cronbach alpha = 0.78).

##### Adolescent behavior composite

To capture whether adolescents respond to their peer context in a for better and worse manner, we used a behavior composite score computed from the Prosocial Tendencies Measure and Adolescent Risk-Taking Scale. Average prosocial tendencies scores were reverse coded, and the (reverse-coded) prosocial tendencies and adolescent risk-taking scores were standardized and averaged to compute a behavior composite score that describes the balance of prosocial and risk-taking behaviors. Lower scores indicate greater endorsement of more prosocial than risk-taking behaviors, higher scores indicate greater endorsement of more risk-taking than prosocial behaviors, and scores of 0 indicate relatively equal levels of prosocial and risk-taking behaviors.

### fMRI data acquisition and preprocessing

#### MRI data acquisition

Imaging data were collected using a 3 Tesla Siemens Trio Prisma MRI scanner. The resting-state sequence lasted 8 min. Adolescents were presented with a white fixation cross on a plain black screen and instructed to remain still with their eyes open. Resting-state scans included 240 T2*-weighted echoplanar images (EPI) (time repetition (TR) = 2 s; time echo (TE) = 25 ms; field of view (FOV) = 230 mm; matrix = 92 × 92; voxel size 2.5 × 2.5x3mm^3^; flip angle = 90 degrees; slice thickness = 3 mm; 37 slices). Some participants (*n* = 12) were unable to complete the full scan session, but still provided partially-complete (minimum 4 min) resting state data (M_volumes_ = 183, SD_volumes_ = 45, range_volumes_ = 129–239). All analyses excluding subjects with partially-complete resting state data yielded similar results; thus, we report results from the full sample here. Structural scans were obtained for registration purposes, which consisted of a T2*weighted, matched-bandwidth (MBW), high-resolution, anatomical scan (TR = 4 s; TE = 64 ms; FOV = 230; matrix = 192 × 192; slice thickness = 3 mm; 38 slices) and a T1* magnetization-prepared rapid-acquisition gradient echo (TR = 1.9 s; TE = 2.3 ms; FOV = 230 mm; matrix = 256 × 256; sagittal plane; slice thickness = 1 mm; 192 slices). To maximize brain coverage and minimize signal drop-out, MBW and EPI scans were obtained using an oblique axial orientation.

#### MRI data preprocessing

Preprocessing was performed using *fMRIPprep* 1.2.6–1^40,41^ (RRID:SCR_016216), which is based on Nipype 1.1.7^42,43^ (RRID:SCR_002502).

##### Anatomical data preprocessing

T1-weighted (T1w) images were corrected for intensity non-uniformity (INU) using N4BiasFieldCorrection^44^ (ANTs 2.2.0). A T1w-reference map was computed after registration and INU-correction using mri_robust_template (FreeSurfer 6.0.1)^45^. The T1w-reference was then skull-stripped with antsBrainExtraction (ANTs 2.2.0), using OASIS as the target template. Brain surfaces were then reconstructed using recon-all (FreeSurfer 6.0.1, RRID:SCR_001847)^46^, and the brain mask estimated previously was refined with a custom variation of the method to reconcile ANTs- and FreeSurfer-derived segmentations of the cortical grey-matter through Mindboggle (RRID:SCR_002438)^47^. Spatial normalization to the ICBM 152 Nonlinear Asymmetrical template (version 209c, RRID:SCR_008796^48^ was performed through nonlinear registration with antsRegistration (ANTs 2.2.0, RRID:SCR_004757)^49^, using the brain-extracted version of both the T1w volume and the template. Brain tissue segmentation of cerebrospinal fluid (CSF), white-matter (WM), and grey-matter (GM) was performed on the brain-extracted T1w volume using FAST (FSL 5.0.9, RRID:SCR_002823)^50^.

##### Resting state data preprocessing

The following preprocessing was performed on the resting-state data. First, a reference volume and its skull-stripped version were generated using custom methodology through *fMRIPrep*. The blood-oxygen-level-dependent (BOLD) reference was then co-registered to the T1w reference created previously using bbregister (FreeSurfer), which implements boundary-based registration^51^. Co-registration was configured with 9 degrees of freedom to account for distortions remaining in the BOLD reference. Head-motion parameters with respect to the BOLD reference (transformation matrices and six corresponding rotation and translation parameters) were estimated before spatiotemporal filtering using MCFLIRT (FSL 5.0.9)^52^. BOLD runs were then slice-time corrected using 3dTshift from AFNI (02/07/2016,RRID:SCR_005927^53^. The BOLD time-series were then resampled to surfaces on the *fsaverage5* space. The BOLD slice-time corrected time-series were resampled to their original, native space by applying a single, composite transform to correct for head-motion and susceptibility distortions, as well as to the MNI152NLin2009cAsym standard space.

To additionally control for head motion artifacts, several confounding time-series were calculated based on the preprocessed BOLD time-series: three region-specific global signals, FD, and DVARS. The three global signals were extracted within the CSF, WM, and whole-brain mask. FD and DVARs were calculated for the resting-state data, using their implementations in *Nipype* (following the definitions of^54^). Volume-to-volume FD of > 0.30 mm were scrubbed (*M*_volume_ = 7.2%, range_*volume*_ = 0–52.9%), resulting in acceptable levels of motion on average (*M*_*FD*_ = 0.12 mm, *range M*_*FD*_ = 0.06-0.20 mm; maximum_*FD*_ = 0.29 mm, range maximum_FD_ = 0.17-0.30 mm). Consistent with rsfMRI studies in developmental samples^6,55^, and given the stringent thresholds employed for scrubbing, we did not exclude further subjects based on the percentage of scrubbed volumes but controlled for the number of usable resting-state volumes in analyses.

Additionally, a set of physiological regressors were extracted to allow for component-based noise correction (*CompCor*)^56^. After high-pass filtering was applied to the preprocessed time-series (using a discrete cosine filter with a 128 s cut-off), principal components were estimated for the two *CompCor* variants: temporal (*tCompCor*) and anatomical (*aCompCor*). Six *tCompCor* components were then calculated from the top 5% variable voxels within a mask covering the subcortical regions. This subcortical mask was obtained by heavily eroding the brain mask, ensuring that it does not include cortical GM regions. For *aCompCor*, six components were calculated within the intersection of the aforementioned subcortical mask and the union of the CSF and WM masks calculated in T1w space following their projection to the native space of each functional run (using the inverse BOLD-to-T1w transformation). The head-motion estimates calculated in the correction step were also placed within the confounds file generated by *CompCor*.

All resamples were performed with a single interpolation step by the composition of all the pertinent transformations (i.e., head-motion transform matrices, susceptibility distortion corrections, and co-registrations to anatomical and template spaces). Gridded (i.e., volumetric) re-samplings were performed using antsApplyTransforms (Advanced Normalization Tools (ANTs)), configured with Lanczos interpolation to minimize the smoothing effects of other kernels^57^. Non-gridded (i.e., surface) re-samplings were performed using mri_vol2surf (FreeSurfer).

### Data analysis

#### Region of interest (ROI) definition

To assess intrinsic network connectivity, we defined region of interest (ROI) masks for four a priori brain networks implicated in social decision making^7^ (Table 1): the mentalizing network, the cognitive control network, the motivational relevance network, and the affective salience network (see NeuroVault^58^: https://neurovault.org/collections/SISNGRAB/). We defined ROIs using anatomical segmentation for smaller subcortical regions that are easily defined anatomically (e.g., amygdala, ventral striatum). For large cortical regions that perform multiple different functions (e.g., dmPFC, TPJ), we further constrained the ROI based on additional functional information (e.g., from NeuroSynth or the Saxe lab mask). Mentalizing regions included bilateral temporoparietal junction (TPJ) defined from the Saxe Lab^59^, bilateral posterior superior temporal sulcus^4^, posterior cingulate cortex, bilateral temporal poles (SPM Anatomy Toolbox^60^), as well as the dorsomedial prefrontal cortex (dmPFC) derived from Neurosynth^61^ by searching the term “dmPFC” in the automated meta-analysis tool and downloading the relevant mask. Because Neurosynth meta-analytic maps can include regions that simply co-occur with the search term, we masked the resulting map with the medial frontal gyrus from the Wake Forest University (WFU) Pickatlas^62^ to remove co-occurring regions. Cognitive control regions included bilateral dorsolateral prefrontal cortex (dlPFC) (WFU Pickatlas) and bilateral inferior parietal lobe (IPL) (SPM Anatomy Toolbox). Motivational regions included the bilateral ventral striatum and bilateral caudate (Harvard-Oxford Atlas), ventral tegmental area^63^ and orbitofrontal cortex (Automatic Anatomical Labeling atlas). Finally, affective salience regions included the bilateral amygdala, dorsal anterior cingulate, bilateral anterior insula, and bilateral putamen defined from the Harvard–Oxford Atlas. Masks were evaluated using the Marsbar toolbox in SPM^64^ in order to ensure that ROIs did not contain any voxels that overlapped with another mask.

#### Timeseries extraction and connectivity calculations

To calculate within-network connectivity for the different networks defined above, we extracted timeseries from each ROI mask using tools from Nilearn^65^. All confounds (e.g., motion parameters) were regressed from each timeseries. The fully processed time-series data were averaged within each ROI and then each ROI’s average time-series was correlated with the average time-series for all other ROIs within that network. We then computed the mean correlation strength of edges (i.e., path connections) between all pairs of nodes (i.e., brain regions) within the same network (e.g., affective salience network). Higher correlation values indicated stronger within-network connectivity, with positive and negative values reflecting positive and negative within-network connectivity, respectively. Within-network connectivity for each of the four networks was positive and moderately strong (*r*s = 0.35-0.56; Table 2).

**Table 2:** Descriptives and correlations among study variables.

Variable	1	2	3	4	5	6	7	8
1. Peer norms	1	.41***	− .09	− .11	− .07	.02	.10	− .22*
2. Adolescent behavior		1	.01	− .003	.02	.11	.25*	.13
3. Affective salience connectivity			1	.64***	.42***	.47***	.06	− .33**
4. Motivational relevance connectivity				1	.37***	.30**	.25**	− .25*
5. Mentalizing connectivity					1	.48***	− .02	.14
6. Cognitive control connectivity						1	− .14	− .09
7. Age							1	.20
8. Usable rsfMRI volumes								1
M	2.17	− .003	.49	.30	.51	.53	12.83	215.66
(SD)	(.72)	(.75)	(.18)	(.15)	(.13)	(.15)	(.53)	(34.66)
Range	1–4.13	− 1.28–2.38	.16–.89	.02–.68	.09–.83	.01–.79	11.94–14.49	92–240

***p < .001; **p < .01; *p < .05. Adolescent Behavior refers to a composite measure computed from the Prosocial Tendencies Measure and Adolescent Risk Taking Scale.

### Analysis plan

We conducted analyses to examine whether findings were consistent with a differential susceptibility model. To support this model, several criteria need to be met: (1) there should be a significant interaction between peer context (i.e., peer group norms) and intrinsic connectivity predicting adolescent behavior, suggesting moderation by neurobiological sensitivity; (2) the association between peer group norms and behavior should be significant in adolescents with high but not low sensitivity; (3) within unfavorable peer contexts (i.e., negative peer group norms), reports of risk-taking behavior should be significantly higher in adolescents with high than low intrinsic connectivity; and (4) within favorable peer contexts (i.e., positive peer group norms), reports of prosocial tendencies should be significantly higher in adolescents with high than low intrinsic connectivity^66^.

To test criterion (1) and (2), hierarchical multiple regression analyses were conducted to examine whether within-network connectivity moderated associations between peer group norms and adolescent behavior. Analyses were conducted in SPSS (version 25, IBM) using the PROCESS macro^67^. Bootstrap bias-corrected confidence intervals (95%) are estimated, where nonzero overlapping confidence intervals indicate a significant effect. All variables were standardized prior to analysis. Adolescent behavior, ranging from prosocial to risky, served as the dependent variable. Age, sex, and the number of usable resting state volumes were entered as covariates. Predictors included peer group norms, within-network connectivity, and their interaction. Given we tested interactions with each network in four separate hierarchical models, we applied a Bonferroni-corrected threshold of *p* < 0.0125 to correct for multiple comparisons (*p* = 0.05/4 comparisons = 0.0125). A significant interaction would suggest that within-network connectivity moderates the effect of peer group norms on adolescent behavior. To probe significant interaction effects, we conducted simple slope analyses using small multiples (created with the R-based interActive data visualization tool)^68^ to test whether the association between peer group norms and adolescent behavior was significant in adolescents with higher but not lower levels of within-network connectivity. In addition, we conducted a supplemental analysis with all four networks entered simultaneously into a separate hierarchical regression model to test whether any of the individual networks was a stronger predictor of peer influence susceptibility (Supplemental Information).

To test criteria (3) and (4), we used the Roisman approach which includes significance testing and the implementation of three indices to assess whether differential susceptibility is supported^66,69^. First, a test of the regions of significance (RoS) examines the values with respect to peer group norms at which the differences between high versus low levels of overall within-network connectivity are significant. If the association between the moderator and the outcome is significant at both the low and high ends of the distribution of the independent variable within the normative range (i.e., ± 2 SD), there is evidence of differential susceptibility. Second, the Proportion of Interaction (PoI) captures the proportion of the entire interaction that reflects a for better and for worse outcome for participants. Although there are no clear cut-offs, PoI values should be between 0.40–0.60^66^, or even a wider range of 0.20–0.80^69^, on either side of the crossover of regression lines in an interaction plot, with an ideal index closer to 50% of the interaction supporting both for better and for worse outcomes. And third, similar to the PoI, the proportion affected (PA) captures the proportion of individuals in the sample who fall above the crossover point for the interaction. This value is an estimate of the proportion of youth who experience the benefits of positive peer group norms. To support differential susceptibility, this value should be above 16%, with an ideal index around 50%.

## Results

Table 2 presents correlations among all study variables. Peer group norms were correlated with adolescent behavior, such that exposure to relatively more negative peer group norms was related to greater endorsement of risk-taking than prosocial behaviors, and exposure to relatively more positive peer group norms was related to greater prosocial than risk-taking behaviors. We found age and sex differences in adolescent behavior, such that older adolescents were relatively more risky than prosocial (see Table 2), and girls tended to be relatively more prosocial (*M* = − 0.15, *SD* = 0.74), whereas boys tended to be relatively more risky (*M* = 0.19, *SD* = 0.73), *t*(85) = 2.17, *p* = 0.03). We therefore controlled for age and sex in analyses.

Primary analyses tested whether intrinsic connectivity within a priori social cognitive and affective networks render adolescents differentially susceptible to peer influence. To test criterion (1) of the differential susceptibility to peer influence hypothesis, we examined whether the interaction between peer group norms and within-network connectivity significantly predicted adolescent behavior, suggesting moderation by neurobiological sensitivity. After controlling for sex, age, and usable resting state volumes as covariates in the first step, we entered the main effects of peer group norms and within-network connectivity in the second step, and their interaction term in a third step. To examine the contributions of each individual network, intrinsic connectivity within each individual network was entered as a moderator in four separate hierarchical multiple regression models. Only intrinsic connectivity within the affective salience network moderated the link between peer group norms and adolescent behavior, as indicated by a significant two-way interaction between peer group norms and within-network intrinsic connectivity (Table 3). Interaction effects with the mentalizing network and cognitive control network trended in the same direction at more liberal thresholds (*p*s < 0.09), and the motivational relevance network was non-significant.

**Table 3:** Intrinsic networks moderate associations between peer group norms and adolescent behavior.

	Affective salience network		Motivational relevance network		Mentalizing network		Cognitive control network	
Predictors	B (SE)	95% CI	B (SE)	95% CI	B (SE)	95% CI	B (SE)	95% CI
*Step 1*								
Sex	− .15 (.07)	[− .25, .03]	− .15 (.07)	[− .26, .04]	− .15 (.07)	[− .25, .04]	− .12 (.07)	[− .23, .06]
Age	.17 (.07)	[− .02, .27]	.14 (.08)	[− .05, .26]	.16 (.07)	[− .02, .27]	.19 (.07)	[− .01, .29]
Usable rsfMRI Volumes	.20 (.08)	[− .01, .30]	.21 (.08)	[− .01, .32]	.12 (.08)	[− .07, .25]	.18 (.08)	[− .02, .28]
*Step 2*								
Peer Norms	.41 (.07)**	[.17, .45]	.40 (.08)**	[.14, .46]	.40 (.07)**	[.15, .45]	.41 (.07)**	[.16, .45]
Connectivity	.09 (.08)	[− .08, .22]	.05 (.08)	[− .13, .21]	− .002 (.07)	[− .15, .14]	.09 (.07)	[− .08, .22]
*Step 3*								
Peer Norms $\times$ Connectivity	.29 (.07)*	[.08, .34]	.08 (.07)	[− .08, .20]	.18 (.06)	[− .02, .24]	.17 (.06)	[− .02, .23]
∆R^2^	.08*		.01		.03		.03	
Adjusted R^2^	.31**		.22**		.24**		.25**	
AIC	− 75.85		− 64.80		− 67.19		− 68.67	

**p < .001; *p < .0125 (Bonferroni corrected). ∆R^2^ = Change statistic of adding Peer Norms $\times$ Connectivity interaction above main effects. *AIC* Akaike information criterion.

A separate hierarchical multiple regression model that simultaneously included all four networks further supported that only the interaction between peer group norms and intrinsic connectivity within the affective salience network was significant after controlling for each network (Table S1). To identify whether intrinsic connectivity within the affective salience network was a stronger predictor of peer influence susceptibility than the other networks, we compared the strength of moderation effects between the affective salience network and each of the other intrinsic networks. Pairwise equality of coefficient (Wald) tests revealed that the moderation effect of the affective salience network was significantly stronger than that of the mentalizing network (*z* = 2.86, *p* < 0.05), cognitive control network (*z* = 3.01, *p* < 0.05), and motivational relevance network (*z* = 3.64, *p* < 0.05). These results demonstrate that intrinsic connectivity within the affective salience network may play a key role in moderating adolescents’ susceptibility to peer influence above and beyond the other intrinsic networks.

**Figure 1:**
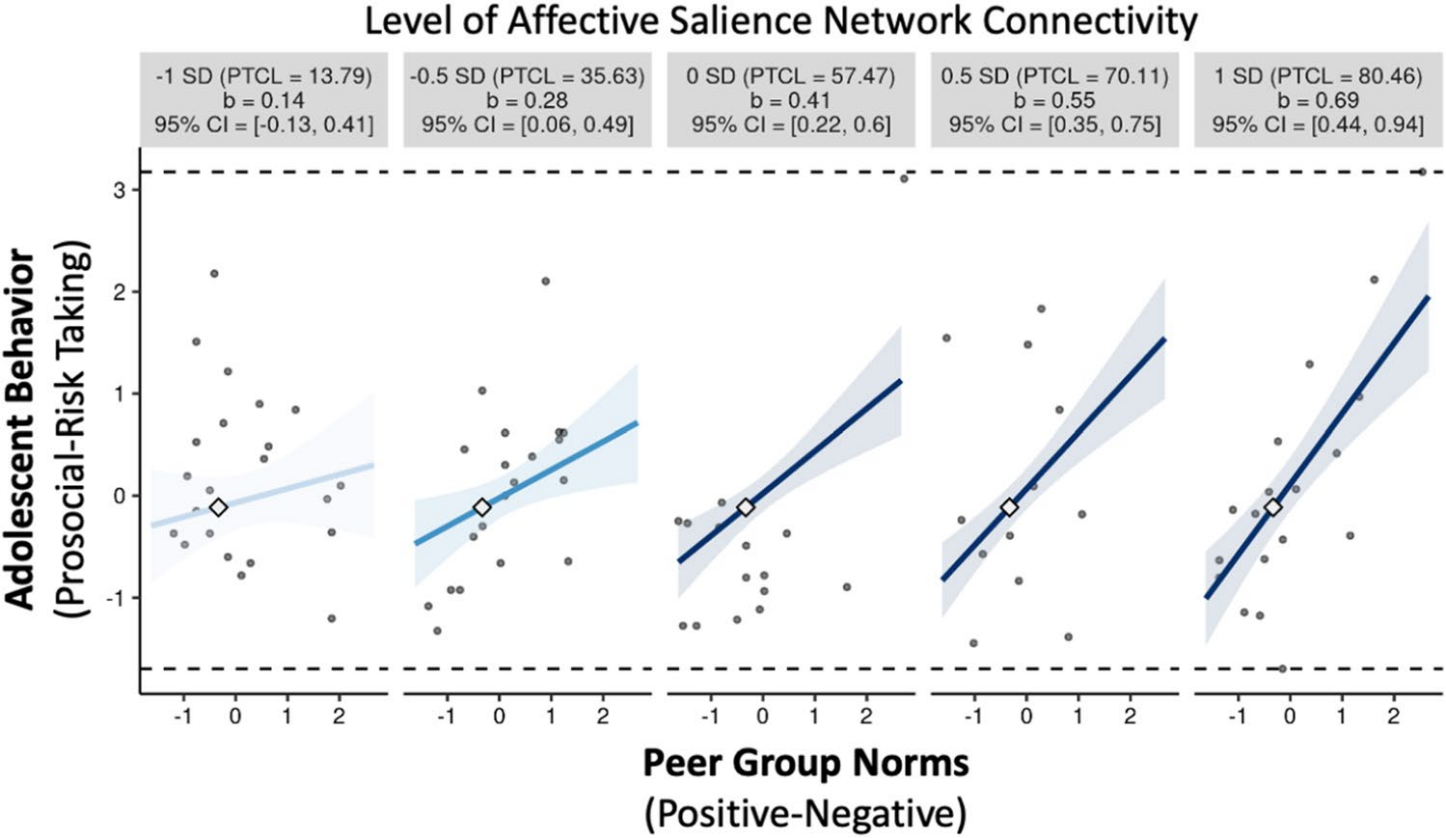
Affective Salience Network Moderates the Association between Peer Group Norms and Adolescent Behavior. The association between peer group norms and adolescent behavior is plotted at five levels of within-network connectivity (range: − 1 SD to 1 SD from mean in .5 SD increments) for the affective salience network. Simple slopes that significantly differ from 0 are shown in dark blue, whereas simple slopes that do not significantly differ from 0 are shown in light blue. Peer group norms significantly predicted adolescent behavior for individuals with higher (.5 SD and 1 SD above mean) and moderate (0 SD; i.e., average) but not lower (i.e., .5 SD or 1 SD below mean) levels of intrinsic connectivity within the affective salience network. *SD* standard deviation; *PTCL* percentile; *CI* confidence intervals.

Given only the moderation effect of the affective salience network was significant across models, remaining tests of the differential susceptibility hypothesis focused on the affective salience network. To test criterion (2), we probed the significant interaction effect within the affective salience network using small multiples to plot a broad range of simple slope effects (i.e., ranging from 1 SD below the mean to 1 SD above the mean in 0.5 SD increments), which displays the observed data that is most representative of each simple slope^68^. Decomposition of the interactions above revealed that peer group norms significantly predicted adolescent behavior for those with higher (0.5 SD or 1 SD above mean) and moderate (0 SD, i.e., average), but not lower (0.5 SD or 1 SD below mean) levels of intrinsic connectivity within the affective salience network (Fig. 1). In other words, the association between peer norms and adolescent behavior was significant in adolescents with high and average, but not low intrinsic connectivity.

**Figure 2:**
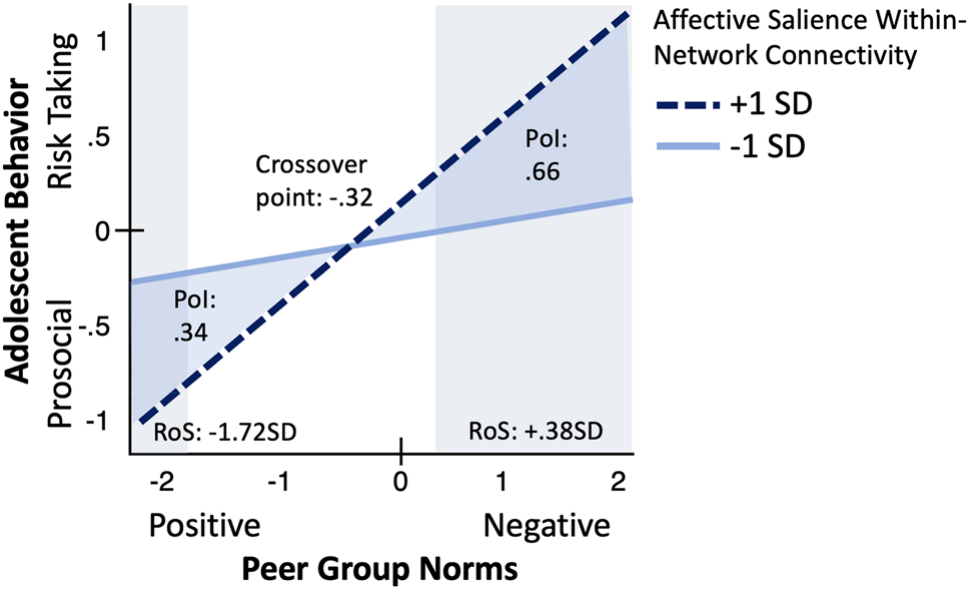
Evidence of Differential Susceptibility to Peer Context. For adolescents with high intrinsic connectivity within the affective salience network (1 SD above mean), those perceiving negative peer norms reported heightened risk-taking behavior whereas those perceiving positive peer norms reported heightened prosocial tendencies. In contrast, adolescents with low intrinsic connectivity within the affective salience network (1 SD below mean) were resistant to peer norms, such that peer norms were not associated with adolescent behavior. *PoI* Proportion of interaction; *RoS* Region of significance, *SD* standard deviation.

We tested criterion (3) and (4) by probing the interaction at low (1 SD below mean) and high (1 SD above mean) affective salience within-network connectivity. As shown in Fig. 2, and consistent with the small multiples identified above, adolescents with relatively lower (i.e., 1 SD below mean) affective salience within-network connectivity were resistant to peer norms, such that peer norms were not associated with adolescent behavior (*b* = 0.10, SE = 0.10). In contrast, for adolescents with relatively high (i.e., 1 SD above mean) affective salience within-network connectivity, those perceiving more negative than positive peer norms reported relatively higher levels of risk-taking behavior than those with low affective salience within-network connectivity, whereas those perceiving more positive than negative peer norms reported relatively higher levels of prosocial tendencies than those with low affective salience within-network connectivity (*b* = 0.52, SE = 0.10). To formally test whether this supports a differential susceptibility model, we computed the RoS, PoI, and PA. The lower-bound and upper-bound RoS were at − 1.72 SD and 0.38 SD, respectively. The PoI was 66% to the right of the crossover and 34% to the left of the crossover, and the PA was 63%. Collectively, these indices provide support for differential susceptibility to peer influence^66,69^.

Supplemental analyses examining risk-taking behavior and prosocial tendencies as separate outcomes further confirmed that the affective salience within-network connectivity confers increased susceptibility to peer influence on each behavior in the hypothesized direction (Supplemental Information). Specifically, adolescents with high affective salience within-network connectivity reported greater risk-taking behavior in the presence of more negative peer norms and lower risk-taking behavior in the presence of more positive peer norms (Table S2). Similarly, adolescents with high affective salience within-network connectivity reported greater prosocial tendencies in the presence of more positive peer norms and less prosocial tendencies in the presence of more negative peer norms (Table S3).

## Discussion

In the current study, we evaluated whether heightened intrinsic connectivity within a set of networks associated with social cognitive and affective processes renders some adolescents more susceptible to peer influence depending on the valence of the environment. Only intrinsic connectivity within the affective salience network significantly moderated the effect of peer group norms on early adolescents’ prosocial tendencies and risk-taking behavior, above and beyond the other three intrinsic brain networks. Consistent with the differential susceptibility model, adolescents with higher intrinsic connectivity within the affective salience network reported greater prosocial tendencies in the context of relatively more positive than negative peer norms and reported greater risk-taking behavior in the context of relatively more negative than positive peer norms. In contrast, adolescents with lower levels of intrinsic connectivity within the affective salience network were resistant to their peer group norms. Intrinsic connectivity within the mentalizing, cognitive control, and motivational relevance networks were not associated with individual differences in peer influence susceptibility. These data underscore the key role of the affective salience network in moderating adolescents’ sensitivity to peer influence beyond traditional networks implicated in social cognition, self-regulation, and motivational processes.

### The costs and benefits of high affective salience network connectivity for adolescent behavior

Comparisons among the social cognitive and affective intrinsic networks that are hypothesized to contribute to peer influence susceptibility revealed that the affective salience network showed the strongest moderation effect on the association between peer norms and adolescent behavior. Recent work has similarly found that functional connectivity between networks supporting affective salience and the rest of the brain predicts the extent of peer conformity^70^. Our findings further underscore affective salience processing of the broader peer environment as a potentially key mechanism underlying increased sensitivity to both positive and negative peer influences in early adolescence. Heightened intrinsic connectivity within the affective salience network may reflect generally stronger attentional biases toward affectively salient cues in the environment. Since flexible, adaptive behavior requires selectively and moderately attuning to social cues^30^ while suppressing goal-irrelevant social information^71,72^, youth who exhibit higher levels of affective salience within-network connectivity may be indiscriminately vigilant toward a wider range of peer contexts given their increased socio-emotional significance during adolescence. Over time, this neural sensitivity may render highly susceptible youth especially responsive to both the adverse and supportive effects of their peer environments relative to their less susceptible counterparts. Although here we focused on how general affective salience sensitivity interacts with different peer group norms, future studies should explore how interactions with other adolescent-typical peer experiences that are known to implicate this network, such as victimization^73^ and rejection^74^, may relate to risk-taking and prosocial behavior.

Consistent with the differential susceptibility theory, we observed that high levels of intrinsic connectivity within the affective salience network rendered adolescents more likely to endorse both risky and prosocial behaviors when the peer context promoted those respective behaviors. Among early adolescents with high intrinsic functional connectivity within the affective salience network, negative peer norms predicted greater frequency of risk-taking behaviors relative to prosocial tendencies. This aligns with previous research showing that even (mis)perceptions of deviant peer norms encourage riskier health behaviors during adolescence^75^, especially among those with a lower ability to resist peer influence^76^. Because engaging in some deviant or health risk behaviors is a normative part of healthy adolescent development, heightened susceptibility to negative peer norms may be more likely to confer significant health problems should engaging in those risk-taking behaviors escalate to dangerous levels or persist into adulthood. Importantly, high intrinsic connectivity within the affective salience network confers developmental benefits when early adolescents are exposed to favorable peer environments, such that those perceiving relatively more positive than negative peer norms reported greater prosocial relative to risk-taking behavior. Thus, despite serving as a potential liability in negative environments, heightened intrinsic connectivity within the affective salience networks may help susceptible youth thrive in positive environments by enabling them to navigate their dynamic social contexts in more flexible ways than their less susceptible counterparts.

### Low affective salience network connectivity is associated with lower susceptibility to peer influence

Previous research has often highlighted the importance of cognitive control mechanisms in reducing adolescent susceptibility to peer influence^77,78^. Here, we show that lower intrinsic connectivity within a set of regions associated with processing affective salience can also facilitate lower susceptibility to peer influence in early adolescence. These results extend prior work focused on traditional regulatory mechanisms to provide a novel understanding of the role of affective salience processing in facilitating differential sensitivity to both the negative and positive effects of peer influence in early adolescence. Given the affective salience network’s role in developing vigilance for detecting social-emotional cues of potential reward or threat, early adolescents with low intrinsic connectivity within the affective salience network may selectively encode such peer-related cues or experiences, rendering them less susceptible to peer norms regardless of whether they perceive frequent prosocial or risky behaviors among their peers. One alternative explanation of these results is that they reflect desensitization to local peer group norms (e.g., unaffected by deviant vs. school norms) rather than a lower sensitivity to the broader peer environment. Although often discussed as a protective factor against negative peer norms, being less susceptible to peer influence may be suboptimal in favorable peer environments, wherein the same peer contagion processes that give rise to unfavorable outcomes can be leveraged to reinforce adaptive behaviors.

### Contributions of other intrinsic brain networks to peer influence susceptibility

Contrary to hypotheses, the mentalizing, cognitive control, and motivational relevance intrinsic networks did not show significant moderation effects in the current study. This is interesting in light of our previous work testing brain-based indices of susceptibility to peer influence, which found that perceived negative peer norms predicted risk-taking behavior only among adolescents with higher VS activity to social rewards and punishments^36^. Given the VS is part of the motivational relevance network, the extent to which the VS responds in a task-specific manner (e.g., to social rewards) may play a more important role in moderating peer influence susceptibility than an intrinsic network of regions broadly specialized for encoding the motivational relevance of the peer context at rest. Indeed, a meta-analysis of fMRI studies of social decision making in adolescents found that the recruitment of several regions within the motivational, mentalizing, and cognitive control networks is sensitive to context-dependent differences in the type of social outcomes, social influence, and actors under consideration^7^. Moreover, to our knowledge, we are the first to compare the contributions of multiple social cognitive and affective mechanisms to peer influence susceptibility, demonstrating that peer influence susceptibility in early adolescence may be shaped more strongly by general attentional biases to affectively salient cues in the environment than the context-dependent processes underlying social information processing, self-regulation, or motivational value.

### Limitations and conclusions

Limitations of this study should be noted. First, given regional brain activity can reflect multiple psychological processes^79^, we cannot conclude that intrinsic connectivity within these four networks at rest directly maps onto changes in affective, motivational, mentalizing, and cognitive functioning in response to external social stimuli. Future research should employ longer rsfMRI scans and general functional connectivity methods that leverage shared features between resting-state and task-based fMRI data^80^ to improve the reliability of intrinsic connectivity measures for predicting individual differences in adolescent outcomes. Furthermore, using independent component analysis- or structural equation model-based approaches to compute data-driven intrinsic brain networks (e.g., Group Iterative Multiple Model Estimation^81^) may offer additional insight into whether these results are robust across functional connectivity metrics or implicate other brain regions or network configurations underlying peer influence susceptibility. Second, we assessed prosocial tendencies across a range of situations, which, despite its stability across contexts, may be limited in their correspondence to actual prosocial behaviors. However, prior work shows strong convergent validity between the PTM and global prosocial behavior^82^. Finally, despite the relatively large sample size, the current study focused on a narrow age range that is limited to early adolescence. While we demonstrate promising links between intrinsic connectivity measures and individual differences in susceptibility to peer influence during this period of heightened social sensitivity, our cross-sectional study cannot speak to whether intrinsic network connectivity precedes peer influence susceptibility, or is merely a concurrent correlate of those who are susceptible to peer influence during early adolescence. Future longitudinal work should address this question, as strong moderating effects of intrinsic connectivity between peer influence and prosocial and risk-taking behaviors may be specific to early adolescence, partially due to puberty and differential maturation of limbic and prefrontal brain systems.

In conclusion, the differential susceptibility theory helps to redress the field’s overemphasis on brain-based vulnerabilities by suggesting heightened neurobiological sensitivity alone is neither “good” nor “bad,” but may instead render some youth more susceptible to certain behavioral outcomes, depending on the type of peer environment encountered. The current findings highlight the costs and benefits of high intrinsic connectivity within the affective salience network to peer contexts for adolescent behavior. By improving the quality of the peer contexts that adolescents are exposed to, we may increase the socialization of positive over negative norms at a time when neurodevelopmental mechanisms render some youth especially receptive to peer influence.

## Supplementary Information

Supplementary Information.

## Data Availability

The ROI masks generated for analyses are available on NeuroVault^58^: https://neurovault.org/collections/SISNGRAB/). The raw data are not publicly available as they contain information that could compromise the privacy/consent of participants, but are available upon request from the corresponding author [EHT] for research purposes only.
