# Supplementary material for: Intrinsic connectivity within the affective salience network moderates adolescent susceptibility to negative and positive peer norms
Source: Sci Rep. 2022 Oct 19;12:17463. doi: 10.1038/s41598-022-17780-1 (PMC9582022; doi:10.1038/s41598-022-17780-1)
Supplement: Supplementary file 1 — Supplementary Information. [file 41598_2022_17780_MOESM1_ESM.docx]

Supplemental Information

Intrinsic Connectivity Within the Affective Salience Network Moderates Adolescent Susceptibility to Negative and Positive Peer Norms

Kathy T. Do, Ethan M. McCormick, Mitchell J. Prinstein, Kristen A. Lindquist, & Eva H. Telzer*****

Results

**Network Contributions to Peer Influence Susceptibility**

In a separate hierarchical regression model, we explored whether each individual network moderated the association between peer norms and adolescent behavior after controlling for each other network. After controlling for sex, age, and usable rsfMRI volumes as covariates in the first step, we entered peer group norms and all four individual networks (mentalizing network, cognitive control network, motivational relevance network, and affective salience network) in the second step. All possible two-way interactions between peer group norms and each of the four networks were entered in a third step. We found that only the interaction between peer group norms and intrinsic connectivity within the affective salience network was significant after controlling for each network (Supplemental Table 1).

**Affective Salience Within-Network Connectivity Moderates Association between Peer Norms and Risk Taking**

In supplemental analyses, we examined whether the interaction between peer group norms and affective salience within-network connectivity predicted each behavioral outcome separately. First, we examined risk-taking behavior as the dependent variable. After controlling for sex, age, and the number of usable rsfMRI volumes as covariates in the first step, we entered the main effects in the second step, and the interaction term in a third step to predict risk-taking behavior. Results revealed a significant main effect of age, significant main effect of peer group norms, and trending interaction between peer norms and affective salience within-network connectivity (Table S2). In exploratory analyses, we probed this interaction at low (1 SD below mean) and high (1 SD above mean) levels of affective salience within-network connectivity. For adolescents with relatively lower (i.e., 1 SD below mean) affective salience within-network connectivity, peer norms were associated with greater risk-taking behavior (*b*=.35, SE=.14, *t*(82)=2.2, *p*=.03). For adolescents with relatively higher (i.e., 1 SD above mean) affective salience within-network connectivity, peer norms was more strongly associated with risk taking (*b*=.67, SE=.12, *t*(82)=4.96, *p*<.001), such that they reported greater risk-taking behavior in the context of negative peer norms, but less risk-taking behavior in the context of positive peer norms. The lower-bound RoS was -.42 SD, while the upper-bound RoS fell outside the range of the data. The PoI was 86% to the right of the crossover and 14% to the left of the crossover, and the PA was 81%.

**Affective Salience Within-Network Connectivity Moderates Association between Peer Norms and Prosocial Tendencies**

Next, we examined (reverse-coded) prosocial tendencies as the dependent variable, such that higher values indicate lower prosocial tendencies. After controlling for sex, age, and the number of usable rsfMRI volumes as covariates in the first step, we entered the main effects in the second step, and the interaction term in a third step to predict prosocial behavior. Results revealed a significant interaction between peer norms and affective salience within-network connectivity (Table S3). We next probed this interaction at low (1 SD below mean) and high (1 SD above mean) levels of affective salience within-network connectivity. For adolescents with relatively lower (i.e., 1 SD below mean) affective salience within-network connectivity, peer norms were not associated with prosocial behavior (*b*=-.16, SE=.16, *t*(80)=-1.01, *p*=.32). In contrast, for adolescents with relatively higher (i.e., 1 SD above mean) affective salience within-network connectivity, peer norms were significantly associated with prosocial behavior (*b*=.37, SE=.14, *t*(80)=2.58, *p*=.01), such that they reported greater prosocial behavior in the context of positive peer norms and lower prosocial behavior in the context of negative peer norms. The lower-bound and upper-bound RoS were at -1.49 SD and 1.24 SD, respectively. The PoI was 49% to the right of the crossover and 51% to the left of the crossover, and the PA was 50%.

**Supplemental Table 1.** Affective Salience Network Moderated the Association between Peer Group Norms and Adolescent Behavior

| *Predictors* | *Adjusted R^2^* | *B (SE)* | *p* | *95% CI* |
| --- | --- | --- | --- | --- |
| Step 1 | .08* |  |  |  |
| Sex |  | -.10 (.07) | .31 | [-.22, .07] |
| Age |  | .18 (.08) | .09 | [-.02, .29] |
| Usable rsfMRI Volumes |  | .19 (.09) | .11 | [-.04, .33] |
| Step 2 | .22*** |  |  |  |
| Peer Norms |  | .47 (.08) | <.001 | [.19, .51] |
| Affective Salience Connectivity |  | -.02 (.11) | .88 | [-.24, .20] |
| Motivational Relevance Connectivity |  | .09 (.11) | .49 | [-.14, .29] |
| Mentalizing Connectivity |  | -.10 (.09) | .40 | [-.25, .10] |
| Cognitive Control Connectivity |  | .10 (.09) | .41 | [-.11, .26] |
| Step 3 | .30*** |  |  |  |
| Peer Norms $\times$ Affective Salience Connectivity |  | .46 (.11)* | .003 | [.12, .55] |
| Peer Norms $\times$ Motivational Relevance Connectivity |  | -.25 (.10) | .08 | [-.38, .02] |
| Peer Norms $\times$ Mentalizing Connectivity |  | .06 (.08) | .67 | [-.13, .19] |
| Peer Norms $\times$ Cognitive Control Connectivity |  | -.01 (.08) | .95 | [-.16, .15] |

****p*<.001; **p*<.05. AIC (Akaike information criterion)=-69.22

**Supplemental Table** **2***.* Affective Salience Within-Network Connectivity Moderates Association between Peer Group Norms and Risk Taking

| *Predictors* | *B (SE)* | *p* | *95% CI* |
| --- | --- | --- | --- |
| Step 1 |  |  |  |
| Sex | -.05 (.09) | .63 | [-.23, .14] |
| Age | .17 (.10) | .08 | [.02, .36] |
| Usable rsfMRI Volumes | .17 (.11) | .11 | [-.04, .38] |
| Step 2 |  |  |  |
| Peer Norms | .51 (.10)*** | <.001 | [.32, .70] |
| Affective Salience Connectivity | .14 (.10) | .18 | [-.06, .34] |
| Step 3 |  |  |  |
| Peer Norms $\times$  Affective Salience Connectivity | .16 (.09) | .077 | [-.02, .34] |
|  |  |  |  |
| ∆R^2^ | .03 |  |  |
| Adjusted R^2^ | .30*** |  |  |
| AIC | -23.21 |  |  |

*Notes:* ****p*<.001. ∆R^2^ = Change statistic of adding Peer Norms $\times$ Connectivity interaction above main effects. AIC = ﻿Akaike information criterion.

**Supplemental Table 3***.* Affective Salience Within-Network Connectivity Moderates Association between Peer Group Norms and Prosocial Tendencies

| *Predictors* | *B (SE)* | *p* | *95% CI* |
| --- | --- | --- | --- |
| Step 1 |  |  |  |
| Sex | -.18 (.11) | .09 | [-.39, .03] |
| Age | .08 (.11) | .47 | [-.14, .29] |
| Usable rsfMRI Volumes | .13 (.12) | .29 | [-.11, .36] |
| Step 2 |  |  |  |
| Peer Norms | .11 (.11) | .34 | [-.11, .32] |
| Affective Salience Connectivity | -.002 (.11) | .99 | [-.23, .23] |
| Step 3 |  |  |  |
| Peer Norms $\times$  Affective Salience Connectivity | .27 (.10)* | .01 | [.06, .46] |
|  |  |  |  |
| ∆R^2^ | .07* |  |  |
| Adjusted R^2^ | .09* |  |  |
| AIC | -2.23 |  |  |

*Notes:* **p*<.05. Prosocial Tendencies is reverse-coded, such that higher values indicate lower prosocial tendencies. ∆R^2^ = Change statistic of adding Peer Norms $\times$ Connectivity interaction above main effects. AIC = ﻿Akaike information criterion.
